# Computational Molecular Phenotypic Analysis of PTPN22 (W620R), IL6R (D358A), and TYK2 (P1104A) Gene Mutations of Rheumatoid Arthritis

**DOI:** 10.3389/fgene.2019.00168

**Published:** 2019-03-07

**Authors:** Noor Ahmad Shaik, Babajan Banaganapalli

**Affiliations:** ^1^Department of Genetic Medicine, Faculty of Medicine, King Abdulaziz University, Jeddah, Saudi Arabia; ^2^Princess Al-Jawhara Center of Excellence in Research of Hereditary Disorders, King Abdulaziz University, Jeddah, Saudi Arabia

**Keywords:** deleterious mutations, rheumatoid arthritis, molecular analysis, protein modeling, biological network

## Abstract

Rheumatoid arthritis (RA) is a chronic autoimmune disorder of bone joints caused by the complex interplay between several factors like body physiology, the environment with genetic background. The recent meta-analysis of GWAS has expanded the total number of RA-associated loci to more than 100, out of which approximately ∼97% (98 variants) loci are located in non-coding regions, and the other ∼3% (3 variants) are in three different non-HLA genes, i.e., TYK2 (Prp1104Ala), IL6R (Asp358Ala), and PTPN22 (Trp620Arg). However, whether these variants prompt changes in the protein phenotype with regards to its stability, structure, and interaction with other molecules, remains unknown. Thus, we selected the three clinically pathogenic variants described above, as positive controls and applied diverse computational methods to scrutinize if those mutations cause changes in the protein phenotype. Both wild type and mutant protein structures of PTPN22 (W620R), IL6R (D358A), and TYK2 (P1104A) were modeled and studied for structural deviations. Furthermore, we have also studied the secondary structure characteristics, solvent accessibility and stability, and the molecular interaction deformities caused by the amino acid substitutions. We observed that simple nucleotide predictions of SIFT, PolyPhen, CADD and FATHMM yields mixed findings in screening the RA-missense variants which showed a ≥*P*-value threshold of 5 × 10^−8^ in genome wide association studies. However, structure-based analysis confirms that mutant structures shows subtle but significant changes at their core regions, but their functional domains seems to lose wild type like functional interaction. Our findings suggest that the multidirectional computational analysis of clinically potential RA-mutations could act as a primary screening step before undertaking functional biology assays.

## Introduction

Rheumatoid arthritis (RA) is a chronic, progressive and disabling autoimmune disease of bone joints (Fazal et al., 2018). Although the full etiology of RA remains unclear, it is widely seen as a complex disease caused by the combined influence of several factors like body physiology, environment and genetic background (Chaudhari et al., 2016; Angelotti et al., 2017). The genetic basis of RA is well demonstrated by epidemiological studies which reported variable disease prevalence’s among different ethnic groups, inheritance pattern in families, and shared susceptibility loci with other autoimmune diseases (Ek et al., 2014). The heritable nature of RA is assessed to be approximately 60%, while the contribution of the human leucocyte antigen region (HLA), which allows the immune system to differentiate between self and foreign bodies, to heritability is estimated to be between 11 and 37%. Out of all HLA genes, the HLA-DRB1 region is recognized as a major contributor of RA susceptibility, i.e., ∼30% (Knevel et al., 2017). In addition to the HLA region genes, some non-HLA candidate genes like PTPN22 and PAD14 have also been discovered to play a major role in RA susceptibility (Rodriguez-Elias et al., 2016).

As in other complex diseases, the advent of Genome Wide Association Studies (GWAS) have dramatically enhanced the resolution of RA genetics, by successfully identifying disease associated common genetic variants (Manolio, 2010). More notably, GWAS followed by subsequent meta-analysis of GWAS data derived from the single nucleotide polymorphisms (SNPs) based dense genotyping of 73,758 controls and 29,880 RA cases, belonging to Asian and European ethnicities, has expanded the total number of RA-associated loci to more than 100 (Okada et al., 2014). This finding has expanded the list of non-HLA loci genes (including PADI4, PTPN22, TNFAIP3, IRF5, STAT4, TRAF1/C5, REL, and CCR6 genes etc) proposed in the genetic etiology of RA. Together, these loci can share the heritability of RA among different ethnicities by ∼80%. Approximately 97% (98 lead SNPs) of these risk loci are located in non-coding regions, and the other 3% (3 SNPs) are found in three different genes, i.e., TYK2 (rs34536443), IL6R (rs2228145), and PTPN22 (rs2476601), which play a critical role in mediating autoinflammation. However, the specific structural and functional deformations caused by these non-synonymous (nsSNPs) is not yet fully explored.

The SNPs which occur in protein coding regions are often evolutionarily conserved and are significant to disease etiology, by changing the amino acid sequence and physicochemical properties of the polypeptide chains they encode. It is therefore possible that these coding SNPs may possess the largest impact in disease pathogenesis, compared to their counterparts, i.e., non-coding SNPs which are mostly neutral to disease pathogenesis (Ng and Henikoff, 2006). Although, both *in vitro* and *in vivo* experimental methodologies can improve our ability to explore the effects of nsSNPs, they often require vast technical resources and manpower in addition to incurring huge costs. In recent decades various computational methods have been launched for predicting the impacts of mutations on protein structure and function. These methods can successfully identify several structural and stability changes which occur in proteins due to mutations (Anwer et al., 2015; Sneha and Doss, 2016; Mohajer et al., 2017). The aim of this study was to assess the ability of diverse computational methods in predicting the missense variant induced alterations in amino acid sequence conservation, structural and functional features of RA candidate proteins. Therefore, in the conext of the current study’s objective, we selected PTPN22 (W620R,) IL6R (D358A), and TYK2 (P1104A) as “positive control variants” because we know their pathogenic potential in contributing to the development of RA, from GWAS findings (≥*P*-value significance threshold of 5 × 10^−8^).

## Methodology

### Selection of RA Susceptibility Loci

The 101 RA susceptibility loci, as confirmed by Okada, Jostins et al. (2012), were used as the core data set for this study. The relevant information (e.g., rsID, Genome wide significance with a ≥*P*-value threshold of 5 × 10^−8^, odd ratios) of these SNPs was retrieved from the article. Only missense mutations were studied in the present investigation. The full-length amino acid sequence of the candidate proteins was collected from UniProt. The description terms like variants, SNPs, and mutations are interchangeably used in this manuscript.

### *In silico* Assessment of Deleterious Potential of nsSNPs of RA

The Variant Effect Predictor (VEP) toolset, hosted on the Ensembl webpage, was used for the analysis and annotation of coding region genomic variants. This powerful computational tool offers accessibility to an extensive range of genetic annotations and provides simple options for configuring and extending the data analysis. VCF (variant call format) is an accepted format of input data for VEP. However, other variant identification formats of dbSNP and HGVS are also recognized by this toolset. The output consists of comprehensive information about mutation (chromosomal location, variant effect, transcript ID) and its functional impact on the protein in form of prediction scores for different computational methods. In this study, the prediction scores of Sorting Tolerant from Intolerant (SIFT) (Vaser et al., 2016). Polymorphism Phenotyping v2 (PolyPhen-2); (Adzhubei et al., 2013), Combined Annotation Dependent Depletion (CADD) (Rentzsch et al., 2018); and Functional Analysis through Hidden Markov Models (FATHMM) (Shihab et al., 2013) for each variant query were generated. The consequences of variants are described in accordance to the standard annotation terms of Sequence Ontology (Cunningham et al., 2015).

### Structural Analysis of RA Proteins

#### 3-Dimensional Modeling of Wild Type and Mutant Protein Models

In the initial stage, the protein data bank (PDB) database was searched for x-ray crystallized structures of candidate proteins. Wherever full length protein structures were not available, we simulated them through *ab initio* procedures using I- TASSER (Iterative Threading ASSEmbly Refinement) web server (Yang et al., 2015), by providing an amino acid sequence of the corresponding candidate protein as an input. This computational tool utilizes basic protein structure templates available in PDB for constructing the full-length atomic models of three-dimensional proteins following the iterative template fragment assembly approach (Yang and Zhang, 2015). Out of the top five models generated for each candidate protein, the best models’ selection was based on the estimated template model (TM) score, confidence score, and root mean square deviation (RMSD) scores. In the eventual stages, these selected models were further processed for energy minimization using the gromacs-steepest descent energy minimization method in NOMAD-Ref web server (Lindahl et al., 2006). The energy minimized three-dimensional protein models were further used in constructing the mutant versions of proteins, by substituting the amino acid residues in place of the reference amino acid sequence of each candidate protein, through a homology modeling procedure using Modeller v9.21 (Webb and Sali, 2017). This software adopts an automated approach for comparative protein structure modeling, through the satisfaction of spatial restraints in the candidate protein of interest (Sanchez and Sali, 2000). The three-dimensional protein structure modeling procedure starts with the alignment of a target model against similar known protein structures. The energy minimization and stereochemical quality check of built mutant models was done using the Procheck tool (Laskowski et al., 1996). Finally, mutant models were visulized by PyMol (Janson et al., 2017) and Chimera computational programs (Banaganapalli et al., 2016; Shaik et al., 2018).

#### Secondary Structure Analysis

The correlation between mutant amino acid sequences and protein structures can be made unambiguous by obtaining information about the alterations of different secondary structural elements such as the orientation and numbers of helices (α), strands (β), bends and turns, which are basic structural elements of protein scaffolds. To obtain the knowledge of the structural context of the amino acid substitution variants of RA-candidate proteins, we used an online web server program called PDBsum. The input for this tool is a PDB structure four-letter code of queried proteins and the output is in the form of a ‘wiring diagram,’ which shows the important secondary structural elements and their orientation along the query protein’s sequence.

#### Solvent Accessibility Analysis

Solvent accessibility refers to the exposed surface area of amino acid residues within a three-dimensional structure and in an extended tripeptide conformation. The ratio of relative surface accessibility of mutated amino acids of RA candidate proteins towards solvents was predicted using the NetSurfP server (Petersen et al., 2009). The data input for this web server consists of a FASTA format of amino acid sequences of the queried protein. The relative solvent accessibility of each amino acid is predicted in the form of a *Z*-score (Klausen et al., 2019).

#### Protein Stability Analysis

DUET, an easy-to-use bioinformatics web server, which integrates two complementary methods such as, mCSM (mutation Cutoff Scanning Matrix) and SDM (Site Directed Mutator) into a consensus/optimized prediction, using Support Vector Machines (SVMs), was used to understand the effect of point mutations on the protein structure. Input options for this tool consists of a PDB structure of a wild-type protein, in addition to amino acid information (wild type or mutant) in one letter codes. The output is in form of individual and combined predictions, in addition to the interactive visualization of proteins. The prediction results are expressed as Gibbs Free Energy (ΔΔ*G*) values, where negative values indicate that the given mutation is highly destabilizing (Pires et al., 2014).

#### Protein Structural Equivalency Analysis

We have superimposed the wild type proteins on to mutant protein structures, to examine structural equivalency of native versus mutant amino acid residues and their corresponding whole protein structures, using PyMOL software (Janson et al., 2017). The input options for superposition requires PDB files (txt format) or PDB accession numbers of both wild and mutant protein structures. The outputs of a structural alignment are a superposition of the atomic coordinate sets (Ca traces and backbone atoms) and a minimal RMSD between the structures. The difference in RMSD scores, of two aligned amino acid residues (<0.2 Å) or whole protein structures (<2.0 Å), indicates their divergence from one another.

#### Conserved Domains Identification

The functional domains in RA candidate proteins were searched in the Conserved Domain Database using query sequences (nucleotide or amino acids) to gain insights into the relationships between mutated amino acid sequence, with both protein structure and function. This tool relies on RPS-BLAST, which can quickly scan the pre-computed position-specific scoring matrices (PSSMs) in the query protein, to identify the different conservation features related to protein domains. The output is in the form of annotated protein domains against the input query sequence, along with visualization options. The high level associations between query protein sequences and annotated conserved domains are shown as specific hits (Marchler-Bauer et al., 2011).

### Gene Functional Analysis

The STRING^1^ web interface, which generates data on gene functions, gene list analysis, and gene prioritization for functional biology assays, was used to identify direct and indirect genetic interaction networks of RA candidate proteins. The input options include either query genes or protein names, their sequences, or large data sets (Szklarczyk et al., 2017). The output format is in the form of network node predictions, which reveal the functional associations between query genes based on their neighborhood or evidence of co-expression in data generated from high-throughput biological experiments. The highest interacting gene or proteins were identified based on the strength of the confidence score generated in the network.

### Protein–Protein Molecular Docking

The Hex protein docking server was employed to execute the molecular docking of RA proteins and interacting proteins, to examine their mutual structural plasticity and interaction potential, while forming molecular complexes. In this step, the protein partner showing the highest confidence score of the queried protein in the molecular network (derived from STRING results), was taken as a ligand molecule and the query protein as a receptor. The molecular models of the experimentally solved crystal structures of proteins retrieved from PDB or *ab initio* modeled protein structures, were used in this step. The major default setting used in the docking procedure is the “free energy calculation,” which is set at 180° to enable the total possible rotational increments for receptor and ligand sampling of their own centroids. The other parameters include a grid spacing set at 0.6 Å, positive and negative steps at 0.75 Å, 53 intermolecular separations calculated in 20 steric scan phases. In the final calculations the final 25 phase was applied to obtain the highest orientation score of 0.76.2 Å. At the end, to obtain 10,000 the lowest ordered docking energy score, 500 clusters were retained from the best 1000 orientations (Ritchie, 2003; Shaik et al., 2014).

## Results

### Coding SNPs of RA Risk Loci

The 101 RA risk loci included 98 (97%) non-coding SNPs and three (3%) coding region SNPs. The molecular details of these three coding SNPs, including chromosomal position, gene name, cDNA change, type and position of amino acid variant are provided in Table 1.

**Table 1:** Basic characteristics of RA-GWAS missense SNPs selected in this study.

Gene symbol	Gene name	rsID	Genomic location	Alleles	cDNA location	Codon change	Exon	Protein effect
PTPN22	Protein tyrosine phosphatase, non-receptor type 22	rs2476601	1:113834946	T/C	c.1858 T > C	Tgg-Cgg	14	Trp620Arg
IL6R	Interleukin 6 receptor (IL6R)	rs2228145	1:154454494	A/C	c.1073 A > C	gAt-gCt	9	Asp358Ala
TYK2	Tyrosine-protein kinase TYK2	rs34536443	19:10352442	C/G	c.3310 C > G	Ccc-Gcc	21	Pro1104Ala

### Deleterious Potential of Missense Mutations

The SIFT prediction algorithm uses the nucleotide sequence homology principle to classify the pathogenic amino acid substitutions, based on their deleterious potential on the function of the concerned protein. SIFT prediction values range from 0 to 1, where lower scores reflect the highly deleterious potential of non-synonymous mutation towards the structural and functional features of the corresponding protein. The missense mutation P1104A (SIFT score is ‘0.’) revealed its highly deleterious potential towards the function of TYK2 protein. The remaining two missense mutations, i.e., W620R of PTPN22 and D358A of IL6 are tolerant to protein’s function (tolerance score index is 0.06 to 1). The Polyphen algorithm uses a Naive Bayes probabilistic score to calculate the pathogenicity potential of amino acid substitution mutations. The P1104A missense mutation of the TYK2 gene showed a probable damaging potential (score is 0.97), whereas W620R of PTPN22 (score is 0) and D358A of IL6 (score is 0.05) are found to be benign in their effect. The CADD is an integrative annotation of multiple mutation prediction methods into one framework. It generates a combined annotation score (*c*-score) for each query variant and classifies them as non-functional variants (if *C-*score is ≥10%), damaging variants (if *C*-score is ≥20%), and lethal (if *C*-score is ≥30%). As per this criterion, only the P1104A variant of TYK2 is a damaging variant. The remaining two variants, i.e., W620R of PTPN22 and D358A of IL6R are non-functional variants with an unknown significance. The FATHMM is a species-independent method, with optional species-specific/evolutionary unit weighting, in order to predict the functional effects of coding variants in form of *P*-values (ranging from 0 to 1) based on ENCODE outcomes. If a given variant shows a *p*-score of ≥0.5 then it is considered as deleterious and if it shows a *p*-score of ≤0.5, then it suggests the neutral nature of the queried genetic variant. Similar to the above three predictions FATHAMM has also predicted the deleterious effect of the P1104A variant on the TYK2 protein (Score is 0.821) (Table 2).

**Table 2:** The SIFT, Polyphen-2, CADD, and FATHMM prediction scores for RA-GWAS amino acid substitution mutations.

No.	Variant	Pathogenicity predictions							
		SIFT		PolyPhen		CADD		FATHMM	
		Score	Prediction	Score	Prediction	Score	Prediction	Score	Prediction
**(1)**	PTPN22-rs2476601	1	Tolerated	0	Benign	10.78	Non-functional	0.028	Benign
**(2)**	TYK2-rs34536443	0	Deleterious	0.97	Probably Damaging	26.1	Damaging	0.821	Deleterious
**(3)**	IL6-rs2228145	0.08	Tolerated	0.05	Benign	4.99	Non-functional	0.163	Benign

### Protein Structural Analysis

#### Protein Modeling and Structural Equivalency Analysis

##### 3D modeling

**FIGURE 1:**
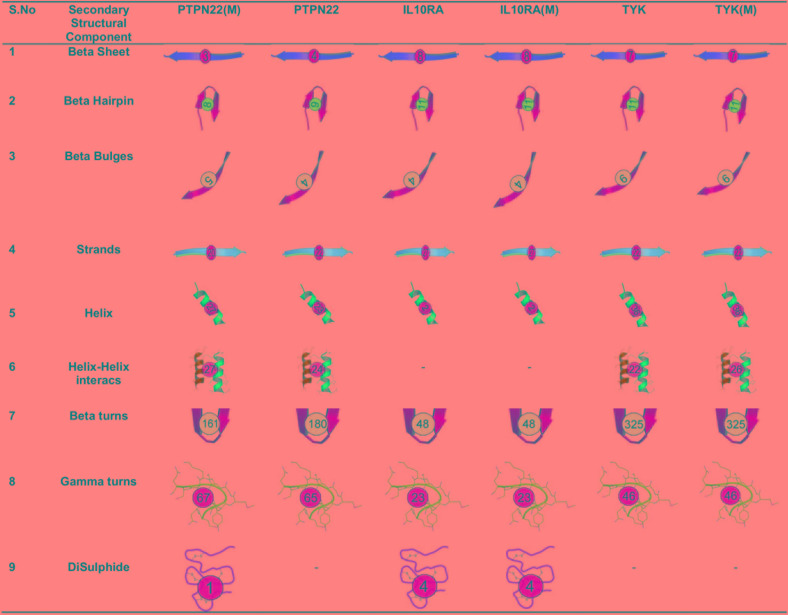
Secondary structure components of wild type and mutant PTPN22, IL10RA, and TYK2.

The I-Tasser prediction server has produced five protein models for each queried amino acid sequence (Uniport ID: PTPN22-Q9Y2R2; TYK2-P29597; IL6R-P08887). From the output, the best protein models were selected based on their TM scores, confidence (c) scores and RMSD scores. The prediction scores of three studied proteins are as follows, PTPN22 (*c* score = −2.12, TM score is 0.46 ± 0.15 and RMSD score is 13.7 ± 4.0 Å), TYK2 (*c* score = −1.12, TM score is 0.26 ± 0.10 and RMSD score is 12.7 ± 2.8 Å) and IL6R (*c* score = −1.65, TM score is 0.35 ± 0.05 and RMSD score is 12.2 ± 3.0 Å). The *C*-score reflects the quality of the predicted protein models and its value ranges from −5 to 2. A higher *C*-score indicates the high-quality of the predicted protein model. A *C*-score of >−1.5 indicates the accurate folding of the polypeptide chain. The full-length models of IL6R, TYK2, and PTPN22 were subjected to energy minimization applying Gromacs 96 force field in Nomad-Ref server (Figure 1). The energy minimization step repairs distorted geometries by moving atoms and releasing the internal constraints in the protein structure. The stereochemical quality checking of full-length models with Procheck software showed that few amino acid residues have their phi/psi angles in the disallowed regions of the protein. Approximately 98.5% of all amino acid residues in three proteins were in the core (allowed region) and 1.5% were in the non-core (disallowed) region, respectively. We have created the mutant versions of proteins, by manually inserting the amino acid change in the protein sequence and template structures of IL6R, TYK2, and PTPN22 native proteins. Out of the 100 guess structures produced (based on randomized initial models), we have selected the best homologous structures of these mutant proteins for energy minimization, stereochemical quality checks and Ramachandran plot assessments.

##### Secondary structure and surrounding amino acid changes analysis

**FIGURE 2:**
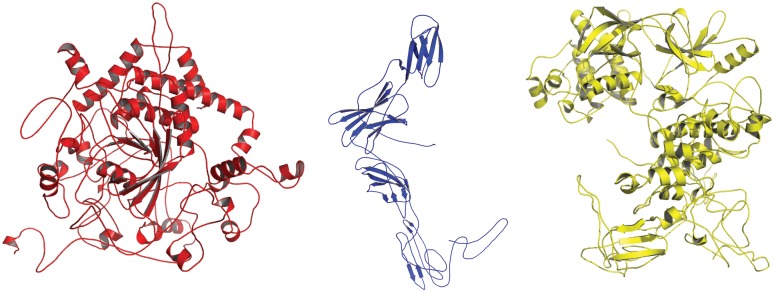
3D-Structural representation of the refined protein models of (1) PTPN22 (red in color); (2) IL6R (blue in color) (3) TYK2 (yellow in color), generated from I-Tasser.

In order to understand the effect of amino acid substitutions on secondary structural features, we calculated the structural differences in elements such as sheets (β-sheets, β-hairpins, β-bulges, and β-strands), helices (helix, helix–helix interactions), and turns (β turns, γ turns, and disulfide bonds) in both native and mutant models of the PTPN22, IL10RA, and TYK proteins (Figure 2). In PTPN22, the substitution of arginine residue at the 620th position generated two β-hairpins, one β-bulge, four helixes, three helix–helix interactions, two γ-turns and one di-sulfide bond and lost one β-sheet and two strands, and 19 β-turns in the secondary structure of the protein. In IL6R (Asp358Ala) and TYK2 (Pro1104Ala) mutant proteins, the numbers of secondary structural elements are observed to be equal like in their native forms. Overall, the structural analysis results inferred that out of three deleterious mutations of RA (PTPN22-Trp620Arg; IL6R-Asp358Ala; and TYK2-Pro1104Ala), only the Trp620Arg (located in loop region) mutant brought drastic changes, disturbing the secondary structure of PTPN22.

##### Root mean square deviation

**FIGURE 3:**
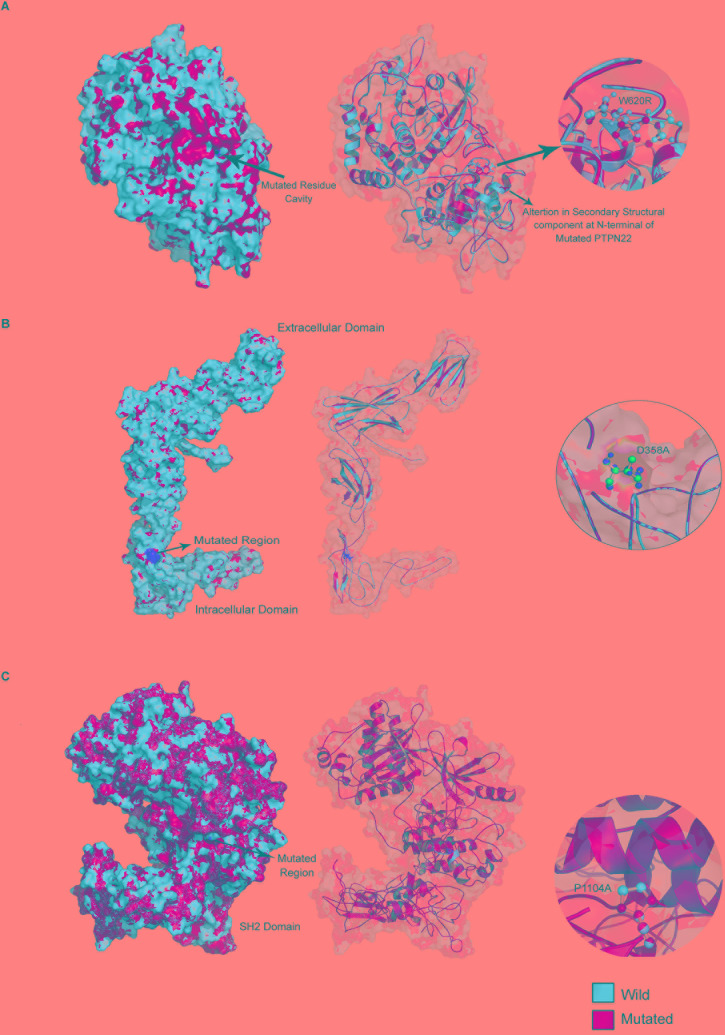
Mutated regions in **(A)** PTPN22, **(B)** TYK2, and **(C)** IL10R proteins.

The structural impact of amino acid substitution mutations can be estimated if there is a divergence at the amino acid or polypeptide chain level of the corresponding proteins. We have observed changes in the cavity volume and residue RMSD scores of Arg620 (2.85 Å) compared to Trp620 of PTPN22. For IL6R (D358V) and TYK2 (P1104A) mutations, the residue level changes were 1.89 and 1.24 Å, respectively (Figure 3).

##### C&D: solvent accessibility and stability (SAS) analysis

The Tryptophan to Arginine conversion at the 620th amino acid residue of the PTPN22 protein, increases its solvent accessibility property by 17.20%. In the IL6R protein, the mutated Valine amino acid residue at the 358th position is exposed to solvents by 51.90% compared to its native counterpart Aspartic acid, which exists in a buried state. In the TYK2 protein, both Proline (wild type) and Alanine (mutant) amino acid residues at the 1104th position, remain in the buried state and inaccessible to solvents (Table 3).

**Table 3:** The solvent accessibility (SA), stability and root mean square deviation (RMSD) prediction scores for three RA-GWAS amino acid substitution mutations.

Protein	Variant	SA		Stability prediction			RMSD	
				McSM (Kcal/Mol)	SDM (Kcal/Mol)	DUET (Kcal/Mol)	Polypeptide Chain (Å)	Amino Acid (Å)
PTPN22	W620R	17.20%	B > E	−0.326	4.47	−0.453	0.02	2.85
IL6R	D358V	51.90%	B > E	−0.197	0.01	−0.093	0.05	1.89
TYK2	P1104A	0%	B > B	−1.699	2.74	−1.493	0.05	1.24

Solvent accessibility and stability is one of the decisive factors in protein folding and to further evaluate the protein stability (net balance of forces that determine the native fold of protein structure) we used an integrated computational approach, the DUET method. This DUET analysis provides the consensus output of SDM and mCSM methods in a non-linear regression fashion, based on Radial Basis Function Kernel function. The Gibbs free energy (ΔΔ*G*) change of the PTPN22, W620R (−0.453 Kcal/Mol), IL6R, D358V (−0.093 Kcal/Mol), and TYK2, P1104A (−1.493 Kcal/Mol) missense mutations were found to be in the range of negative values, further indicating their deleterious potential to the stability of the proteins concerned.

#### Conserved Domains Identification

**FIGURE 4:**
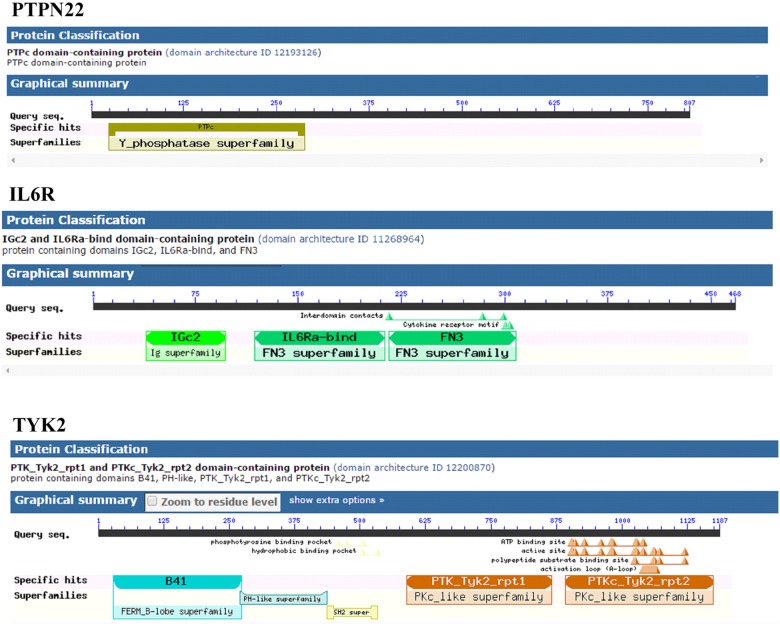
Distribution of conserved domains in PTPN22, TYK2, and IL6R.

The mapping of evolutionary conserved domains of disease-causing proteins, is an essential step in inferring the relationship between the nucleotide sequence and protein structure and its function. Functional annotation of 807 amino sequences of PTPN22 has revealed that the Trp620Arg variant is located outside (332 amino acids downstream) of the protein tyrosine phosphatase (PTP) domain which exists in between the 24th and 288th amino acids. The PTP domain catalyzes the dephosphorylation of phosphorylated tyrosine peptides and regulates its levels in the signal transduction pathways. The Asp358Val of human IL-6R alpha, is located outside of three domains [Ig superfamily, two fibronectin type III (FnIII) domains each ≈100 residues] of the extracellular region of the 468 amino acids long protein molecule. The domain mapping of Pro1104Ala mutation of the TYK2 protein, which is made up of 1187 amino acids, revealed that it was located in the c-terminus pseudo kinase catalytical domain (897–1169) of the TYK2 protein (Figure 4).

### Gene Network Analysis

**FIGURE 5:**
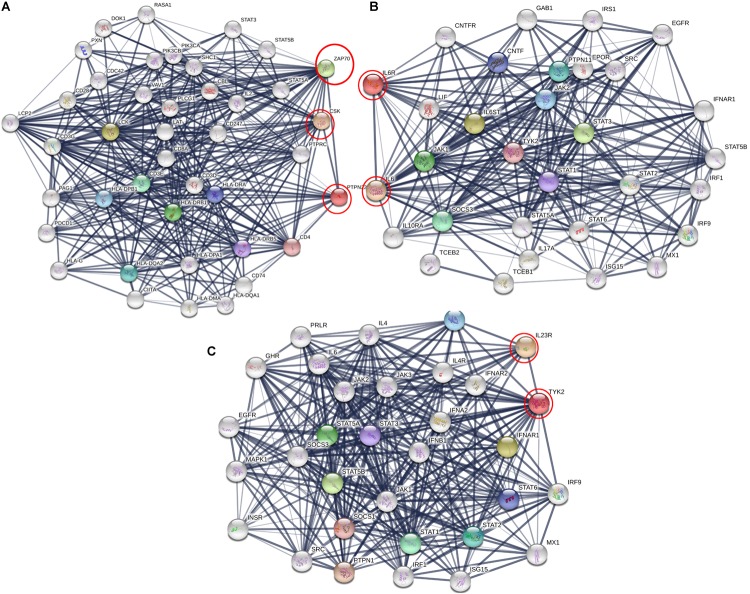
Protein interaction network of **(A)** PTPN22, **(B)** IL6R, and **(C)** TYK2 using STRING web server. Here genes are represented as nodes and edges indicating different types of interaction between genes. Black circles are the query genes and coloring on edges indicate different types of interaction which is defined in the network legend.

We analyzed the genetic association networks of three RA genes, i.e., PTPN22, IL-6RA, and TYK2, to identify their best physical interacting partners in a cellular context. The PTPN22 gene network analysis revealed its tight network with 10 genes including CSK, LCK, ZAP70, HLA-DRB1, CD3E, HLA-DQA2, HLA-DPB1, HLA-DRA, HLA-DRB5, and CD4, of which the highest interaction score (*c*-score = 0.995) was observed with ZAP70. The IL6R protein showed highest interaction with its ligand IL6 molecule (*c*-score = 0.998). It also showed diverse association levels with other networking genes such as IL6ST (0.990), STAT3 (0.989), JAK1 (0.971), SOCS3 (0.971), PTPN11 (0.962), JAK2 (0.957), CNTF (0.952), STAT1 (0.951), and TYK2 (0.942). For the TYK2, the best interacting partner was IL23R, which had a *c*-score of 0.98. Besides these protein molecules, it also interacts with STAT5B, STAT5A, STAT1, STAT2, IL23A, STAT6, STAT3, SOCS1, and PTPN1 proteins (*c*-scores ranging from 0.98 to 0.99). From the predicted interaction networks, it is evident that all three RA genes, in conjunction with their gene partners, activate different auto immune reactions central to the pathogenesis of RA (Figure 5). We also identified the relationship between PTN22, TYK2, and IL6R, using protein–protein network analysis, we identified that TYK2 and IL6R are significantly enriched in the Interleukin-6 signaling and Tyrosine phosphorylation pathways, the hallmark pathway in inflammation (Figure 5).

### Molecular Docking

Based on the results obtained from the protein networking analysis, we identified the best interacting partners of the PTPN22, IL6R, and TYK2 proteins and studied the impact of mutations on their molecular function. Table 3 shows that RA missense mutations contribute to altered interactions with ligand molecules, primarily because of the shift in interacting amino acid residues. Among all the proteins analyzed, TYK2 was calculated to show the lowest binding energy value of −19.90 Kcal/mol with IL23R.

**FIGURE 6:**
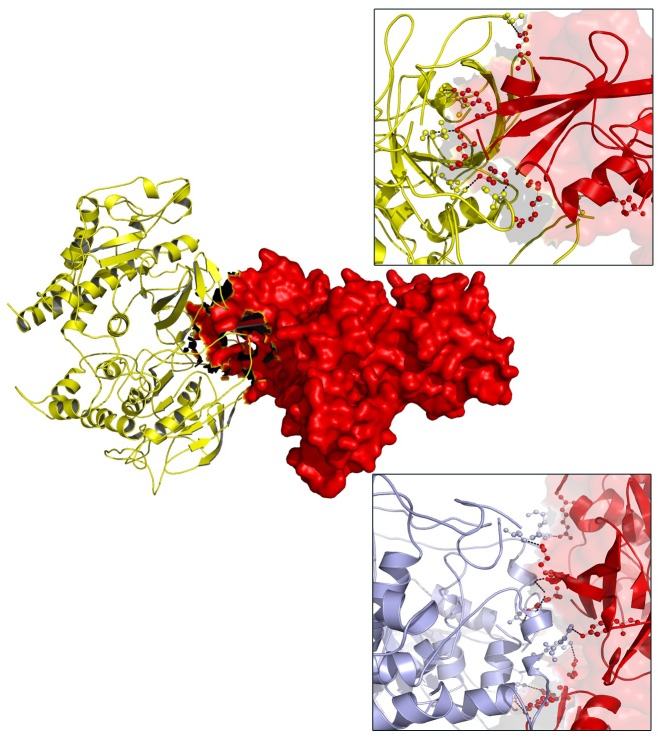
Docking view of PTPN22 (wild type) ZAP70 and PTPN22 (mutated)-ZAP70.

**FIGURE 7:**
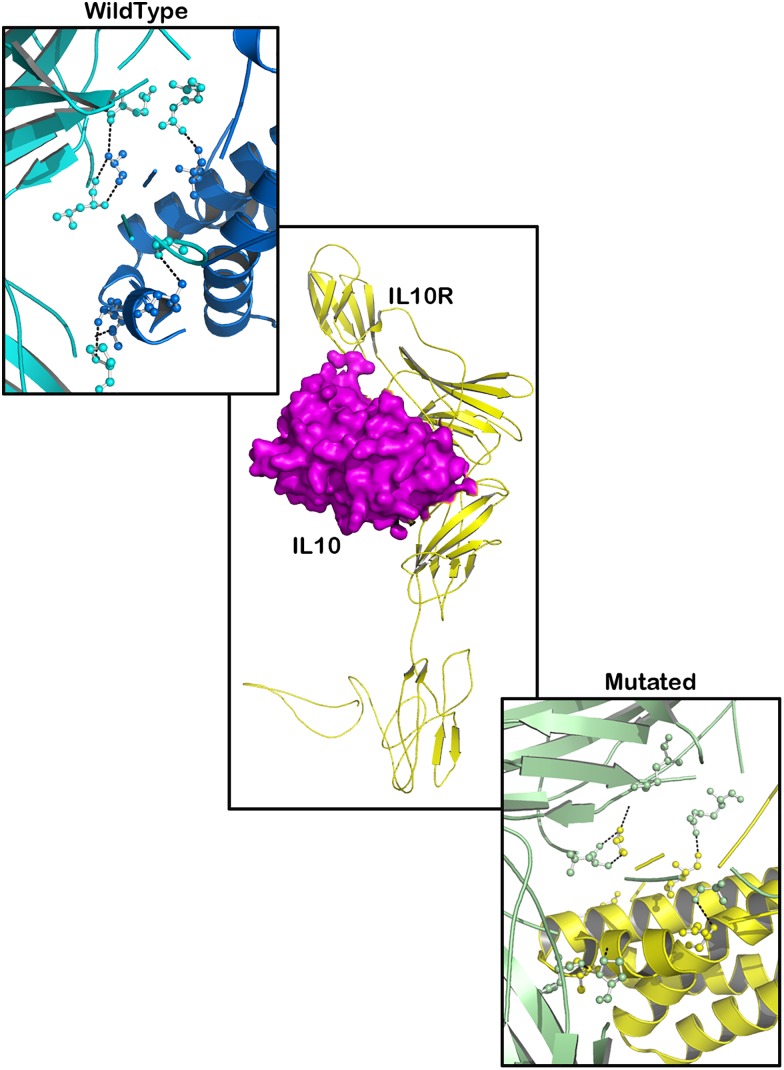
Molecular interaction of IL10R (wild type)-IL10 and IL10R (mutated)-IL10.

**FIGURE 8:**
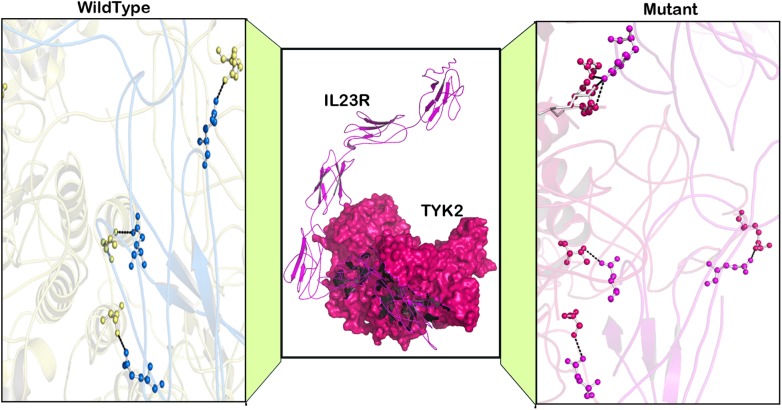
Docking pose of IL23R-TYK2 (wild type) and IL23R-TYK2 (mutated).

TYK2 (P1104A) showed a weaker interaction (−29.25 Kcal/mol) with IL23R. In native TYK2, Arg539, Thr1161, and Ser1157, amino acids form a hydrophobic interaction with IL23, whereas in the P1104A mutant stage, two new Asp949, Arg952 amino acids participate (in addition to Arg539, Thr1161, and Ser1157) in forming strong H-bonds with Asp349 and Arg351 of IL23R. The PTPN22 wild type protein interacts with six amino acid residues (Ala372, Lys683, Asn806, Gln457, Asp388, and Asn432) of the ZAP70 protein with a binding constant of −391.18 KJ/Mol. However, the W620R mutant PTPN22 binds at a different region of ZAP70 (Lys663, Meth729, Lys300, Ser643, Gln307, Thr545, Gly303, and Ser652) due to a change in the conformation of the mutant structure showing a reduced binding affinity (Δ*G* is −407.20 KJ/Mol) towards ZAP70. In case of the native IL10R molecule, the docking analysis revealed that five amino acid residues (Ala7, Ser, 171, Gln118, Arg137, and Pro30) interact with the IL10 ligand molecule, by forming strong Hydrogen, ionic interactions and a binding affinity of −554.7 Kcal/Mol. However, in the D358V mutant, Ala7, Leu12, Gln118, Arg137, Pro308, and Trp306 amino acids participate in forming the majority of the weak interactions with IL10, and positively shifts the interaction energy to +28.17 Kj/Mol (Table 4 and Figure 6–8).

**Table 4:** The molecular docking scores for RA-GWAS amino acid substitution mutations.

Protein complex		Binding energy^a^ (Kcal/Mol)	Energy difference	Interacting amino acids
Protein	Ligand			
Wild type PTPN22(S)	ZAP70(R):	−391.18 KJ/Mol	+16.02 Kj/mol	Ala372, Lys683, Asn806, Gln457, Asp388, Asn432
W620R-PTPN22(S)	ZAP70(R):	−407.20 KJ/Mol		Lys663, Meth729, Lys300, Ser643, Gln307, Thr545, Gly303, Ser652

Wild type IL10(R)	IL10(L)	−554.7 KJ/Mol	+28.17 Kj/Mol	Ala7, Ser, 171, Gln118, Arg137, Pro30
D358V-IL10(R)	IL10(L)	−582.87		Ala7, Leu12, Gln118, Arg137, Pro 308, Trp306

Wild-TYK2(L)	IL23(R)	−19.90	+9.35 Kj/Mol	Arg539, Thr1161, Ser1157
P1104A-TYK2(L)	IL23(R)	−29.25		Arg539, Thr1161, Ser1157, Asp949, Arg952

*^a^The change in binding free energy is related to the inhibition constant as per the following the equation: ΔG = RT in Ki, where R is the gas constant 1.987 cal K^−1^ Mol^−1^, and T is the absolute temperature assumed to be 298.15 K.*

## Discussion

There have been numerous attempts to understand the mechanisms through which RA genetic variants could influence the activity of candidate proteins and subsequently affect the cell physiology and function. For PTPN22, animal model studies have shown that the tryptophan (W) amino acid at the 620th position positively increases the autoreactive B cell selection, the enlargement of the thymus and spleen, T cells, and dendritic cell activation (Stanford et al., 2010; Zhang et al., 2011). In mice, the expression levels of LYP/Pep were found to be reduced when Pep619W (equivalent of W620R variant) was expressed. It is suggested to be due to the calpain1-mediated rapid degradation of the Pep619W variant compared to that of wild types (Menard et al., 2011; Diaz-Gallo and Martin, 2012). The PTPN22/W620R expression enhances the production and function of neutrophils, calcium release and free radical oxygen concentration around joints. The hyperactivation of W620R carrying cells may directly cause damage to bone joints, in addition to the initiation and continuation of other inflammatory reactions that contribute to the disease development (Bayley et al., 2015). The cytokine interleukin-6 (IL-6) is known to regulate the complex mechanisms that underlie the inflammatory reactions of diverse chronic diseases (Ferreira et al., 2013). In RA patients, the IL-6 molecule is overexpressed in inflamed synovial tissues, where it effects the functions of lymphocytes (T and B), macrophages and osteoclasts by binding to IL-6R (Smolen et al., 2008). A genetic polymorphism of the IL-6 receptor locus (Asp358Ala) is known to cause changes in serum IL-6R levels and a modest change in levels of IL-6. Functional studies demonstrated that IL-6,358Ala allele increases the expression of soluble ILR isoform, but reduces the membrane bound (CD4+ T cells and monocytes) isoform, which further results in the impaired IL-6R response (Ferreira et al., 2013). The protein TYK2, which belongs to the Janus kinase superfamily (JAKs), interacts with the intracellular domain portion of the type 1 interferon receptor. Other receptors such as IL-6, IL-10, IL-12, and IFNλ are also known to interact with TYK2 (Ghoreschi et al., 2009). The rs34536443 variant that causes the Proline to Alanine substitution at the 1104th amino acid, occurs in the αFG helix of TYK2. Experimental studies observed that the TYK2, P1104A variant influences the enzyme activity of the protein and thereby also the associated cytokine pathways (Li et al., 2013). The modified TYK2 activity may exacerbate T helper cell polarization across bone joints in individuals that carry the RA associated AlaTYK2 allele.

Recent decades have seen a surge in the discovery of different variant filtering computational methods to analyze the GWAS data (Hou and Zhao, 2013). Most of these better performing methods align multiple protein sequences and judge the functional relevance of residues based on their evolutionary conservation across different species. Detailed comparisons of different variant prediction methods (SIFT, Polyphen, CADD, and FATHMM) and their prediction sensitivities are reviewed elsewhere (Cooper and Shendure, 2011; Miosge et al., 2015; Lu et al., 2016). Although, W620R of PTPN22, D358A of IL6R and P1104A of TYK2 variants are known to have very strong genome wide significant associations (≥*P*-value threshold of 5 × 10^−8^) with the RA risk, computational methods like SIFT, Polyphen-2, CADD, and FATHMM have predicted them to be non-deleterious. These findings underline that nucleotide variant prediction methods are not always sensitive in predicting the pathogenic potential of clinically significant variants. This could be due to the differences in datasets used in training these variant prediction programs (Al-Abbasi et al., 2018). We have therefore further investigated the impact of RA mutations on structural features of proteins.

It is noteworthy to mention that disease can arise if missense mutation leads to the loss or gain of critical functions, due to the altered conformation of secondary structures. Secondary structures are the most common energetically favorable polypeptide structures, which further folds to form super secondary structures, domains, motifs and tertiary structures (Khan and Vihinen, 2007). We have investigated the existence, position and distribution of three missense mutations in secondary structures of those corresponding proteins. Overall, the secondary structural analysis results inferred that out of three deleterious mutations of RA (PTPN22-Trp620Arg; IL6R-Asp358Ala; and TYK2-Pro1104Ala), only the Trp620Arg (located in loop region) mutant brought drastic changes that disturbed the secondary structure of PTPN22. The substitution of the arginine residue at the 620^th^ position in PTPN22, resulted in the gain of extra sheets (two β-hairpins and one β-bulge), helices (four helixes and three helix–helix interactions) and turns (two γ-turns and one disulfide bond) and the loss of one β-sheet and two strands. Since changes in the secondary structural elements were observed; we can expect some promising changes in the pattern of hydrogen bonds between amino hydrogen and carboxyl oxygen atoms in the peptide backbone of PTPN22, which may result in the alteration of dihedral angles in the protein structure.

Our three-dimensional protein modeling and structural equivalency analysis observed huge changes in cavity volume and amino residue RMSD scores of Arg620 (2.85 Å) compared to the Trp620 of PTPN22. Following this trend, even IL6R (D358V) and TYK2 (P1104A) variants have also shown similar structural changes. The degree of protein structure difference caused by mutant amino acid residues, in general, corresponds to their biophysical and chemical properties such as charge, size, molecular weight, hydrophobic nature and chemical side chains (Miao and Cao, 2016). These structural aberrations may, in turn, affect H-bond, ionic, and Vander wall interactions required to uphold the secondary (α helix and β-sheets), tertiary (3D) and quaternary (biomolecular complexes) structural features of RA proteins. The specific function of a protein is related to the interaction of its exposed surface with a solvent. Amino acid residues that form the hydrophobic core of a protein are critical for its stability, therefore they are the site of deleterious mutations. Our predictions show that the Arginine residue at the 620th position of the PTPN22 protein, and the Valine residue at the 358th of IL6R, and the Proline residue at the 1,104th of TYK2 changes the physical orientation and solvent accessibility of the concerned protein molecules. A change, whether an increase or decrease in the surface accessible area to solvents, is determined by the physical orientation of the amino acid (exposed or buried), which could in turn affect the tertiary structure of the proteins (Ramsey et al., 2011). The consensus output of the mutation Cutoff Scanning Matrix and Site Directed Mutator methods revealed the negative Gibbs free energy (DDG) changes for W620R of PTPN22, D358V of IL6R and P1104A of the TYK2 mutations. Negative free energy changes (namely the ΔΔ*G* sign) shifts the stability of proteins to destabilization.

Most disease associated mutations not only interfere with the structural conformation and stability of protein structures, but also induce changes in the molecular binding energies (Hijikata et al., 2017). We therefore further studied the impact of three RA missense mutations in the context of intermolecular interactions. Our protein networking analysis of three RA proteins, i.e., PTPN22, IL-6RA, and TYK2 highlighted their functional interlinking with several immune system molecules, reinforcing the centrality of immune dysfunction in the pathogenesis of RA. Our molecular docking results show that RA missense mutations contribute to altered interactions with ligand molecules, primarily because of the shift in interacting amino acid residues. For molecular docking, we used the Hex Sever, which conveniently performs high quality exhaustive rigid body docking predictions. It takes only ∼15 s to perform 6D docking calculations with this server (Macindoe et al., 2010). All three proteins analyzed, PTPN22 (W620R variant) IL6R (D358V variant) and TYK2 (P1104 variant) were predicted to manifest shifts in the interacting amino acid residues and ligand binding energy values with their corresponding ligand molecules. Furthermore, these three SNPs may also indirectly influence the RA development through the expression of the genes located in the regions of their under-linkage disequilibrium.

## Conclusion

In the present study, the difference between variant prediction methods (pathogenicity predictions, 3D protein structure mapping and alterations in molecular interaction abilities) in classifying the three best known RA missense mutations was shown. We noticed that simple nucleotide predictions of SIFT, PolyPhen, CADD, and FATHMM yielded mixed findings when screening the clinically potential variants. The underlying reason for these diverse prediction outcomes could be due to the differences in datasets used in training these variant prediction programs. In fact, a comprehensive multidirectional screening ranging from secondary structure analysis, 3-D modeling, structure equivalency, surface accessibility and stability analysis, protein networking analysis, to molecular docking analysis will provide a more realistic prediction of variant effects. However, the variant prediction test outcomes require validation by functional biology assays.

## Data Availability

Publicly available datasets were analyzed in this study. This data can be found here: https://www.ebi.ac.uk/gwas/.

## Author Contributions

NS and BB: conceptualization, formal analysis, investigation, methodology, resources, supervision, validation, writing original draft, and writing review and editing. BB: data curation, software, and visualization. NS: funding acquisition and project administration.

## Conflict of Interest Statement

The authors declare that the research was conducted in the absence of any commercial or financial relationships that could be construed as a potential conflict of interest.

## Footnotes

1 https://string-db.org/
